# Reduced Graphene Oxide Nanosheet-Decorated Copper Oxide Nanoparticles: A Potent Antifungal Nanocomposite against Fusarium Root Rot and Wilt Diseases of Tomato and Pepper Plants

**DOI:** 10.3390/nano10051001

**Published:** 2020-05-24

**Authors:** Sozan E. El-Abeid, Yosra Ahmed, José-Antonio Daròs, Mohamed A. Mohamed

**Affiliations:** 1 Mycology and Disease Survey Research Department, Plant Pathology Research Institute, Agricultural Research Center, Giza 12619, Egypt; sozanelabeid@yahoo.com (S.E.E.-A.); yosra242@yahoo.com (Y.A.); 2 Instituto de Biología Molecular y Celular de Plantas (Consejo Superior de Investigaciones Científicas—Universitat Politècnica de València), Avenida de los Naranjos, 46022 Valencia, Spain; jadaros@ibmcp.upv.es; 3 Nanotechnology & Advanced Nano-Materials Laboratory (NANML), Mycology and Disease Survey Research Department, Plant Pathology Research Institute, Agricultural Research Center, Giza 12619, Egypt

**Keywords:** agro-nanotechnology, antifungal nanomaterials, reduced graphene oxide, copper oxide nanoparticles, Fusarium root rot, Fusarium wilt, solanaceous plants

## Abstract

Sustainable use of nanotechnology in crop protection requires an understanding of the plant’s life cycle, potential toxicological impacts of nanomaterials and their mechanism of action against the target pathogens. Herein, we show some properties of a candidate antifungal nanocomposite made from copper oxide (CuO; otherwise an essential soil nutrient) nanoparticles (NPs), with definite size and shape, decorating the surface of reduced graphene oxide (rGO) nanosheets. The successful preparation of the rGO-CuO NPs was confirmed by spectroscopic and microscopic analyses, and its antifungal activity against wild strains of *Fusarium oxysporum* affecting tomato and pepper plants was successfully confirmed. A comparative analysis in vitro indicated that this nanocomposite had higher antifungal activity at only 1 mg/L than the conventional fungicide Kocide 2000 at 2.5 g/L. Further investigation suggested that rGO-CuO NPs creates pits and pores on the fungal cell membranes inducing cell death. *In planta* results indicated that only 1 mg/L from the nanocomposite is required to reduce Fusarium wilt and root rot diseases severity below 5% for tomato and pepper plants without any phytotoxicity for about 70 days. Comparatively, 2.5 g/L of Kocide 2000 are required to achieve about 30% disease reduction in both plants. The present study contributes to the concept of agro-nanotechnology, showing the properties of a novel ecofriendly and economic nanopesticide for sustainable plant protection.

## 1. Introduction

Fusarium wilt disease is a major challenge for vegetable production worldwide [1,2]. The disease caused by the pathogenic fungus *Fusarium oxysporum* is characterized by a deadly vascular wilt syndrome in solanaceous plants [3]. The fungus *F. oxysporum* is one of the most destructive soil-borne fungi that infect crops, particularly tomato, eggplant, pepper, and potato, because it has the potential to survive in the soil for more than two decades. Over one hundred and twenty different strains or *formae speciales* of *F. oxysporum* have been described so far, each one highly specific to a particular host in which it causes disease [4]. *F. oxysporum f. sp. lycopersici* (FOL) and *F. oxysporum f. sp. capsici* (FOC) cause Fusarium wilt in tomato and pepper plants, respectively. Also, *F. oxysporum f. sp. radicis lycopersici* (FORL) causes root rot disease, one of the most widespread fungal diseases in most African and Asian Mediterranean countries [5].

Tomato (*Solanum lypersicum* Mill.) is ranked number one among all produced vegetables, accounting for about 14% of the total vegetable production worldwide. In 2005, Heuvelink and Dorais reported that about four million hectares of arable land are cultivated with tomatoes worldwide, producing more than 100 million tons of total production, valued 5–6 billion US$ [6]. Noteworthily, Egypt is ranked among the top 10 tomato growing countries worldwide, producing more than 6.4 million tons on some 181,000 ha [6]. More than two hundred diseases have been reported affecting tomato crops, which account for about 70 to 95% of the worldwide annual losses of this crop. Fusarium wilt disease accounts for 10 to 50% of these losses [7].

Until recently, different management strategies have been applied to reduce Fusarium root rot and wilt disease incidence. For example, organic control, resistant varieties, crop rotation, and soil solarization [8]. Cu-based biocides are considered the most effective compounds against Fusarium diseases like wilt and root rot [9]. However, the long term use of these compounds has induced undesirable pathogen resistance [10]. Besides, their residues in soil and food are detrimental to human beings and the environment [11]. Therefore, there is an urgent need to develop effective and safe alternatives to protect crops from wilt disease caused by *F. oxysporum*. In the past few years, different types of metal oxides and inorganic nanobiocides, such as ZnO [12,13], TiO_2_ [14], and Ag, received much interest as alternative solutions in plant disease management. In particular, antimicrobial agents based on Ag nanoparticles (NPs) were intensively researched because of their excellent antifungal activity [14,15,16,17]. In fact, different studies reported the use of Ag-based nanocomposites loaded on different carrier systems, like graphene oxide (GO) [18], silicon oxide nanospheres, and porous carbon [18]. Although Ag-based nanocomposites exhibited potent antimicrobial activity against different plant pathogens, their high price and some reported phytotoxicity have restricted their use, so the search for a cost-effective and safe alternative has continued. Researchers recently reported the promising antimicrobial properties of Cu NPs [19,20]. Interestingly, in addition to plant defense, Cu NPs have been reported as a nutrient supplement able to improve plant development in *Vigna radiata* and maize [19,20,21]. However, pure Cu NPs are prone to agglomerate, which prevents their antimicrobial action [22]. To address this problem, GO, a material with excellent physicochemical properties, widely exploited in biomedical, environmental, and agricultural applications [23], or even better reduced GO (rGO), a derivative with better properties, has been proposed as a carrier agent to stack CuO NPs, which showed antimicrobial properties against *Pseudomonas syringae* [24]. In this regard, rGO-CuO nanocomposite exhibited excellent antibacterial activity, leading to complete microbe inactivation upon contact [25,26]. However, rGO and CuO NPs that have been independently tested for their antifungal activity against different phytopathogenic fungi [27,28], have never been analyzed in combination. In this work, we produced CuO NPs, loaded on the surface of rGO nanosheets, and analyzed the properties of this nanocomposite to protect tomato and pepper plants from Fusarium wilt and root rot diseases.

## 2. Materials and Methods

### 2.1. Chemical Reagents

Natural graphite powder (200 mesh, 99.9% purity), cupric chloride, concentrated sulphuric acid (98%), potassium permanganate, hydrogen peroxide (30%), hydrochloric acid, hydrazine hydrate (80%) and ammonia solution (30%) were purchased from Nanjing Chemical Reagent Co., Ltd. (Nanjing, China). L-Ascorbic acid (L-AA) was purchased from Sinopharm Chemical Reagent Co., Ltd. (Beijing, China). All chemicals were of analytical grade and used as received without further purification. Deionized water was prepared in a Milli-Q Plus system (Millipore, Milford, MA, USA).

### 2.2. Fungal Strains and Culture Conditions

Tomato and pepper plants showing Fusarium root rot and wilt symptoms were collected during summer 2017. Fungi were isolated from necrotic tissue of tomato and pepper stems. Small sections (3–5 cm long) of tomato and pepper stems showing vascular discoloration were rinsed thoroughly in tap water. After surface-disinfesting in sodium hypochlorite (2%) for 2 min, the stem pieces were extensively washed with sterile water, dried on sterile filter paper and plated onto potato dextrose agar (PDA) medium, amended with streptomycin sulfate (300 mg/L). Fungal cultures were incubated for 14 days at 26 °C. Isolated fungi were identified using a morphological identification key [29,30]. DNA analysis was use for phylogenetic confirmation.

### 2.3. Molecular Identification of Fusarium Strains

Fusarium isolates were grown on PDA for 14 days at 26 °C, and one disk agar plug (5 mm) was taken from the leading edge for each isolate and placed separately in a small flask containing 50 mL of sterile potato dextrose broth (PDB). After incubation at 25 °C for 5 days, the fungal mycelium was filtered through cheesecloth, washed extensively with sterile water and finally transferred to sterile filter paper to remove water excess. Around 100 mg of harvested mycelium was ground to a fine powder in the presence of liquid nitrogen. The ground mycelium was transferred to a 1.5 mL microcentrifuge tube and DNA was extracted using the DNeasy plant mini kit (Qiagen, Hilden, Germany), according to the manufacturer’s recommendations.

Molecular identification of *F. oxypsorum* isolates was performed using the species specific primer pair uni-F/uni-R [30]. Moreover, the identity of *formae speciales* of the tomato isolate (FORL) was determined using primer sprl-f and sprl-r previously described [30]. The PCR mixture and thermal conditions were performed according to [31]. In addition, PCR amplification and sequencing of the internal transcribed spacer (ITS) region of rDNA were also performed for the three *F. oxypsorum* isolates using the ITS1 and ITS4 primers [32]. PCR reactions were performed in a 25 μL final mixture volume containing 2 μL of 10 ng/μL of genomic DNA, 2.5 μL of 10× PCR buffer, 1 μL of dNTPs 10 mM each, 1.5 μL of 25 mM MgCl_2_, 0.5 μL each of forward and reverse primers (0.5 mM) and 0.2 μL of Taq DNA polymerase (5 U/μL; Biomatik LLC, Canada) [31]. The amplification program included an initial denaturalization cycle of 3 min at 94 °C, followed by 35 cycles of 15 s at 95 °C, 30 s at 53 °C, 80 s at 72 °C, and a final extension step of 10 min at 72 °C, in a thermal cycler (Applied Biosystems, USA). After amplification, the PCR products were analyzed by electrophoresis in a 1% agarose gel. PCR products were purified and sequenced using the same forward and reverse primers. ABI trac files were analyzed and contigs were constructed using the BioEdit sequence alignment editor. Experimental sequences of all isolates were deposited in the GenBank.

### 2.4. Preparation of rGO Nanosheets

rGO was synthesized by an in situ chemical co-precipitation method [30]. A representative procedure is as follows: 1.5 g of GO was dispersed in 600 mL of ultrapure water by ultrasonic treatment (150 W) until the preparation became clear with no visible particulate material. Then, 50 mL ammonia solution (30%) was added to bring the solution to pH 10.0, and the mixture was sequentially stirred for 30 min to promote the complete growth of the nanocrystals. The suspensions were cooled down to room temperature, centrifuged, and properly washed, firstly with ethanol followed by washing with distilled water three times. rGO nanosheets were dried in a vacuum oven overnight. In order to obtain rGO with deeper reduction degree, the above described process was slightly modified. Ten mL of hydrazine hydrate (80%) was added under constant stirring for 4 h at 90 °C.

### 2.5. Synthesis and Characterization of rGO-CuO NPs

Firstly, CuO NPs were synthesized via chemical precipitation method; a standard procedure was followed [33]. The synthesis process was optimized under different conditions, producing three different sizes of CuO NPs (5, 20 and 50 nm). Then, briefly, 40 mg of rGO were dissolved in about 40 mL of H_2_O and flaked into a homogeneous suspension by sonication for 30 min. Next, 0.5 g of each size CuO NPs were added into the rGO suspension and stirring continued for 30 more min. One ml of ammonium hydroxide was rapidly added into the mixture while keeping stirring for 1 h. The resulting product was extensively washed with deionized water, centrifuged, and dried at 60 °C for 1 h. The product was finally stored at 4 °C for characterization and further measurements.

A sample solution of rGO-CuO NPs was prepared in distilled water (1 mg/mL), and a drop of this solution was added onto coated copper grids and let it to dry at room temperature. The sample was then analyzed using a TECNAI 10 transmission electron microscope (TEM, Philips, Amsterdam, The Netherlands). Moreover, an elemental analysis was performed with a JEM- 2100F high-resolution TEM (HRTEM, JEOL, Tokyo, Japan), equipped with an INCA energy-dispersive X-ray spectroscopy (EDS) device (Oxford, London, UK). Microstructures were recorded on a MSAL-XD2 X-ray diffractometer (XRD, Bruker, Karlsruhe, Germany) employing Cu target in the 2q range from 5o to 80 o (40 kV, 30 mA, ƛ = 1.54051 3A). The chemical functional groups responsible for the interaction of the rGO nanosheets with the CuO NPs were further investigated by Fourier transform infrared (FTIR) spectrophotometry (Avatar-300, Nicolet, Green Bay, WI, USA).

### 2.6. In Vitro Antifungal Activity of rGO-CuO NPs

The antifungal activity of rGO-CuO NPs was measured on three *F. oxysporum* strains following the agar dilution method. In brief, the agar medium was supplemented separately with three different concentrations (1, 10, 100 mg/L) of each of rGO-CuO NPs of different sizes (5, 20 and 50 nm). A small disc (0.5 cm diameter) of the active mycelial growth from each of *F. oxysporum* strain, taken from the edge of an 8-day-old fungal culture, was placed in the center of each plate. The inoculated plates were then incubated at 26 ± 2 °C for 8 days. The antifungal effect of rGO-CuO NPs was finally evaluated by measuring the radial growth of fungal hyphae in each inoculated plate [31]:

Inhibition (%) = [(R − r)/R] × 100(1)

where R is the radial growth of fungal hyphae on the control plate and r is the radial growth of fungal hyphae on the plate supplemented with the different rGO-CuO NP preparations. The commercial chemical fungicide Kocide 2000 (Certis, Columbia, MD, USA), was used as a positive control. All in vitro experiments were conducted in triplicate under sterile conditions.

### 2.7. Fluorescence Microscopy Imaging

Briefly, 50 mL of fresh conidial suspensions (3 × 10^7^) were treated with 50 mL of rGO-CuO (1 mg/L) at 26 ± 2°C for 1 h and then the mixtures were stained with 10 mL propidium iodide (PI; Sigma-Aldrich, USA; excitation/emission at 535 nm/617 nm) for 15 min. The slide counter was then stained with 10 mL 4’-6-diamidino-2-phenyl-indole (DAPI; Sigma-Aldrich, USA; excitation/emission at 358 nm/461 nm) for 5 min in the dark [34]. Control samples were treated with deionized water. Next, samples were observed under an inverted fluorescence microscope (Eclipse Ti, Nikon, Tokyo, Japan). The cell death (%) was calculated from the ratio of the number of cells stained with PI (dead fungal spores) to the number of total cells, stained with DAPI or PI.

### 2.8. Fungal Cell Morphology Analysis

The morphological changes of the fungal cells and their spores were further investigated using a scanning electron microscope (SEM) after treatment with rGO-CuO NPs. The fungal suspensions were treated with rGO-CuO NPs for 1 h at 26 °C. After centrifugation at 1372 RCF, the condensed fungal spores, were fixed with 2.5% glutaraldehyde, post-fixed with 1% aqueous OsO_4_ (Fluka, Buchs, Switzerland) and washed with 0.1 M, pH 7.0 phosphate buffer. Subsequently, the samples were dehydrated in an ascending ethanol series (30, 50, 70, 80, 90 and 100%) for 10–15 min, and dried in a vacuum oven. Finally, ultrathin sections containing the treated fungal cells were placed on the copper grids and observed under a SEM (JEOL JSM-6700F).

### 2.9. Inoculum Preparation for In Vivo Experiments

Fusarium isolates were firstly grown on half strength PDA for about a week. Inoculum was prepared in sorghum meals. Briefly, about 200 g of sorghum (*Sorghum bicolor* (L.) Moench) seeds were soaked overnight in water in one-quart Mason jars and autoclaved twice. Once the autoclaved sorghum seeds were cooled down, they were inoculated with 8 mycelial plugs containing Fusarium conidial spores from each isolate. The fungal cultures were then allowed to grow on sorghum for 3.5 weeks; then harvested and air-dried. One part of the Fusarium-infested sorghum was then mixed with 10 parts of sterile 1:2 soil: sand mixture to make up the inoculum for the tomato and pepper root infection assay.

### 2.10. Antifungal Analysis In Vivo of rGO-CuO NPs

rGO-CuO NPs were dissolved in H_2_O at 1 and 100 mg/L. Thirty-days-old tomato (cv. TH99806) and pepper (cv. Hyb. Morad) seedlings were gently removed from their wetted soil and placed into 50 mL of rGO-CuO NPs solution (1 and 100 mg/L) for 1 or 3 h. Nine plant replicates for each NP concentration at each incubation time, as well as control conditions, were used. After incubation, seedlings were gently re-transplanted in the infested soil (4–5 seedlings per pot). Positive and negative controls for the experiments consisted of seedlings treated with the chemical fungicide Kocide 2000 and mock-treated with H_2_O, respectively. Plants were cultivated in a greenhouse. The number of tomato and pepper plants showing wilt or root rot symptoms were recorded starting from the tenth day post-inoculation (dpi).

### 2.11. Analysis of Plant Height, Dry Weight and Photosynthetic Pigments

The physiological effects of rGO-CuO NPs on height, dry weight, and photosynthetic pigments level (chlorophyll and carotenoid content) of tomato and pepper plants were also assessed. Plant height and dry weight were measured at 70 dpi. For measuring the pigments level, 100 mg of leaf tissue from each set of treatments, including the mock-treated control, was fractioned in 7 mL dimethyl sulfoxide (DMSO) and incubated at 65 °C until the complete release of chlorophylls and carotenoids into the solution. After the incubation, the absorbance was recorded (Unico UV-2100 spectrophotometer, Unico Instrument Co. Ltd, China) [35]. Absorbance was measured against a blank of 95% ethanol at the three wavelengths of 452, 644 and 663 nm. Chlorophyll and carotenoid concentrations were calculated as mg/g fresh weigh (FW) [36].

### 2.12. Translocation of rGO-CuO Nanoparticles in Tomato and Pepper Plants

Different root samples were collected from both tomato and pepper treated and control plants. The samples were firstly dried in hot air oven at 60 °C for 24 h and then finely grinded and powdered using a sterilized mortar and pestle. About 0.5 g of the powdered samples were slowly mixed with 10 mL concentrated HNO_3_ and digested for 2–3 h. After digestion, the samples were filtered using Whatman filter paper (0.45 µm). The concentrations of rGO-CuO NPs internalized into filtered plant tissues were determined using inductively coupled plasma (ICP) mass spectrometry (ELAN DRC-e, Perkin Elmer, (Santa Clara, CA, USA). After the NP uptake by the root cells, their translocation inside the cell layers was determined by ultrastructure analysis of roots treated with 100 mg/L of rCuO-CuO NPs using TEM. A number of small root tips (2 × 2 × 2 mm in size) taken from both untreated and treated plants were fixed 2.5% glutaraldehyde and 2% paraformaldehyde prepared in 0.1 M sodium phosphate buffer (pH 7.2) for 2 h at normal temperature followed by overnight incubation at 4 °C shaking. The processed sample grids were then visualized and investigated under JEOL 2100F TEM (JEOL, Tokyo, Japan).

### 2.13. Statistical Analysis

One-way analysis of variance (ANOVA) and Duncan’s multiple range test were conducted to confirm the statistical differences between treatment groups by statistical software package Design Expert (Stat-Ease, Inc Minneapolis, MN, USA). All the operated experiments were conducted in triplicate and the obtained data were showed as mean ± standard deviation (SD). The obtained results were considered statistically significant when the p-value was <0.05. Stat-Ease Design Expert v7.0.0 was also used to determine the significant factors among all studied factors.

## 3. Results and Discussion

### 3.1. Characterization of Wild Fusarium Strains

To analyze the antifungal properties of rGO-CuO NPs against *F. oxysporum*, we first isolated three wild strains of this fungus from tomato and pepper plants, which showed wilt symptoms, growing in Giza, Egypt in 2017. The three isolated strains, named FORL (isolated from tomato), FOC1 and FOC2 (isolated from pepper), were firstly identified based on their morphological characteristics. FORL mycelia were slightly white in color on PDA plates, while FOC1 and FOC2 were white to pink, and sometimes they exhibited a deep pink color on the lower side of the culture plate (Figure S1). All three fungal isolates produced single microconidia with ovate to slightly reniform shape with no septum. Their size ranged 7.33–15.10 × 2.26–4.3 µm in the case of FORL and 7.44–14.55 × 2.19–4.9 µm in FOC1 and FOC2. The macroconidia from the three isolates were elliptical with gradually pointed ends having 3 septa as usual. The size of macro-conidia ranged 27.0–45.2 × 2.8–4.8 μm in FORL, and 25.31–42.9 × 2.1–5.3 μm in FOC1 and FOC2 (Figure S1).

### 3.2. Molecular Identification of Fusarium Strains

PCR analyses on the three Fusarium isolates using uni-F/uni-R primer pair gave DNA products with sizes around 670 bp, which confirmed their identity as *F. oxysporum* strains. PCR amplification using primers sprl-f/sprl-r gave a 947-bp-long product in the case of FORL, indicating that this strain, which was isolated from tomato, is actually *F. oxysporum* f. sp. *radicis-lycopersici*. Amplification of the ITS region with primers ITS1/ITS4 yielded 512 to 530-bp DNA products. BLASTn analysis of the sequence of FORL, FOC1 and FOC2 amplification products exhibited e-values of zero and 100% similarity with GenBank *F. oxysporum* accessions no. LR535799.1, MH396480.1 and MK571196.1, respectively, which confirmed morphological characterization. The partial sequences of the ITS regions from *F. oxysporum* FORL, FOC1 and FOC2 isolates were deposited in GenBank with accession nos. MK937617, MK937618 and MK937619, respectively (Figure S2).

### 3.3. Characterization of rGO-CuO NPs

**Figure 1:**
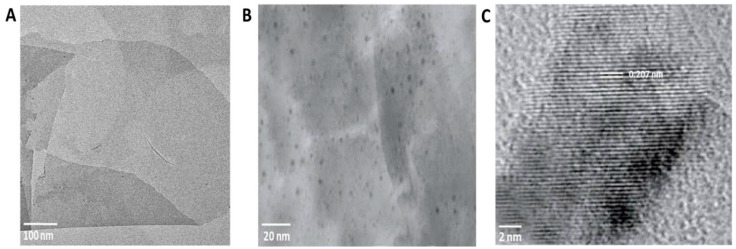
TEM images of (**A**) rGO and (**B**) rGO-CuO NPs. (**C**) EDS pattern of rGO-CuO NPs.

**Figure 2:**
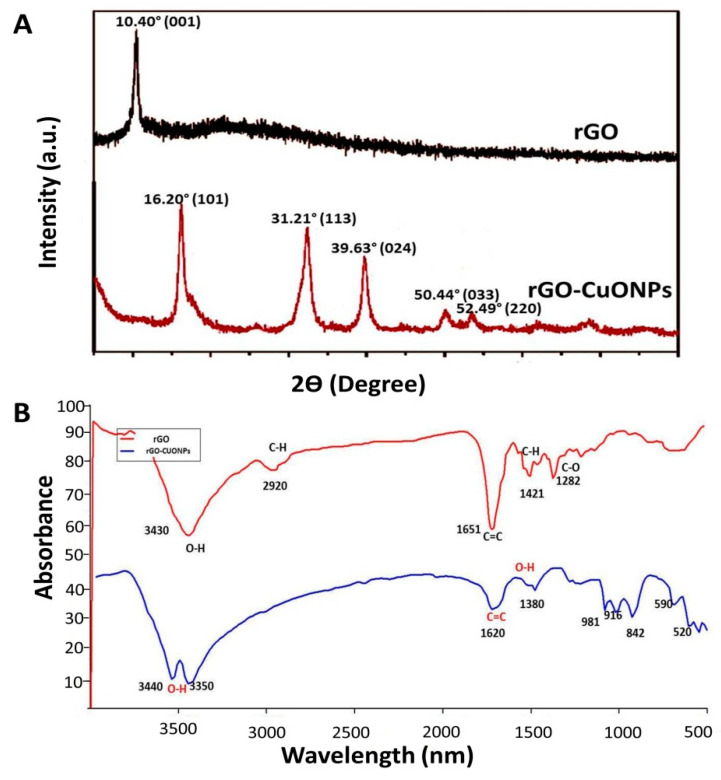
Characterization of rGO-CuO NPs. (**A**) XRD patterns and (**B**) FT-IR spectra of rGO and rGO-CuO NPs, as indicated.

The size of CuO NPs was measured through TEM. Three different sizes (approximately 5, 20 and 50 nm in average) were observed (Figure S3). The different CuO NPs were immobilized individually on the surface of rGO with the aid of an ammonia reductant solution in the presence of a stabilizing agent. The rGO nanosheets can be observed as semitransparent thin sheets in Figure 1A. After the coupling reaction, many dark colored spots, likely CuO NPs, were observed firmly immobilized on the surface of the rGO nanosheets (Figure 1B). EDS analysis showed lattice fringes values of 0.207 nm, which can be attributed to the (111) lattice plane of CuO NPs (Figure 1C). The elemental composition of the produced nanocomposite was determined by EDS and indicated that the rGO-CuO NPs contained carbon, oxygen, chlorine, and copper elements and the copper content accounted for 52.21% (wt) of the nanocomposite. Furthermore, the elemental composition of both rGO and rGO-CuO NPs were further confirmed by XRD. Patterns showed a specific characteristic peak centered at 2Ɵ = 10.40, indicating the high purity and crystalline structure of rGO (Figure 2A). Noticeably, this peak declined in case of rGO-CuO NPs, indicating that CuO NPs altered the structure of the rGO surface. The diffraction peaks of rGO-CuO NPs at diffraction angles of 16.20°, 31.21°, 39.63°, 50.44°, and 52.49° were indexed to the crystalline planes (101), (113), (024), (033) and (220), respectively, referring to the paratacamite crystal (Cu_2_(OH)_3_Cl, JCPDS file no. 87-0679).

The chemical structures of rGO and rGO-CuO NPs were also analyzed by FT-IR to investigate the shifting in chemical functional groups as shown in Figure 2B. FT-IR spectrum for rGO exhibited different peaks of functional groups, including characteristic peaks centered at 3430 cm^−1^ (O-H), 1651 cm^−1^ (C=C), and 1282 cm^−1^ (C–O). Moreover, the characteristic peaks centered at 2920 cm^−1^ and 1421 cm^−1^ were attributed to C–H bonds in –CH3 and –CH2 and the in-plane bending vibration of C–H respectively (Figure 2B). 

Most importantly, several changes between rGO and rGO-CuO NPs were observed in the functional groups. The characteristic peak centered at 3430 cm^−1^ was split into two unique different peaks at stretching vibration 3440 cm^−1^ and 3350 cm^−1^, both of them attributed to O–H [35]. Also, the adsorption peak of C=C at 1651 cm^−1^ was reduced to 1620 cm^−1^ [35]. In contrast, a number of peaks centered at 2920 cm^−1^, 1421 cm^−1^ and 1282 cm^−1^ noticeably disappeared, and others appeared centered at 1380 cm^−1^, 981 cm^−1^, 916 cm^−1^, and 842 cm^−1^. Taken together, these results indicated that CuO NPs were successfully immobilized on the surface of the rGO nanosheets.

### 3.4. Antifungal Activity of the rGO-CuO NPs

**Figure 3:**
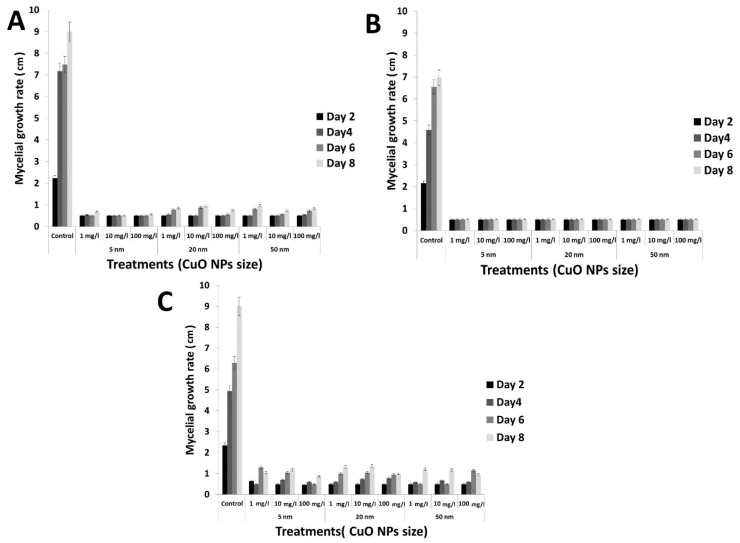
Antifungal activity of rGO-CuO NPs against *F. oxysporum* strains. Plot of fungal growth rate versus CuO NP size and rGO-CuO NP concentration at different time points, as indicated, for FOR) (**A**), FOC1 (**B**), and FOC2 (**C**).

Next, we tested the antifungal activity of rGO-CuO NPs of different sizes (5, 20 and 50 nm) at several concentrations (1, 10 and 100 mg/L) against the three isolated *F. oxysporum* strains in vitro. Results showed that the antifungal activity of the nanocomposite increased as CuO NP size decreased (Figure 3). This observation may be explained by the larger surface area of the smaller NPs compared to their larger counterparts, which could promote more interactions with the fungal cells as well as faster release of Cu ions [37,38]. Interestingly, the statistical analysis showed that the NP size is a significant factor when analyzing the antifungal effect at the different rGO-CuO NP concentrations. Based on this result, we selected the 5-nm rGO-CuO NPs for further work. Table 1 shows the inhibition of mycelial growth for FORL, FOC1 and FOC2 after the different treatments with this 5-nm rGO-CuO NPs.

These results showed that the highest levels of fungal growth inhibition rate (%) against all the three studied isolates of *F. oxysporum* were obtained at 100 mg/L for the smallest NPs (5 nm), with significant values of 94.44 ± 0.0, 92.80 ± 0.33 and 87.8 ± 2.5% for FORL, FOC1 and FOC2, respectively, when compared to a control treatment with a chemical fungicide (68.3 ± 1.1, 68.9 ± 0.4 and 70.5 ± 1.0%, respectively), after eight days of incubation (Table 1). Taken together, these results support that rGO-CuO NPs are a promising alternative to classic fungicides in crop protection. 

This is in agreement with different studies revealing that both rGO and CuO NPs exhibit multiple modes of inhibitory action to microbial pathogens [37], enabling them to be used for controlling a number of microbial pathogens infecting plants, as an alternative to classic chemical pesticides. This would significantly contribute to reduce the hazardous effect of toxic fungicides, especially on edible plants and fresh vegetables [23,39].

### 3.5. Fungal Cell Viability after rGO-CuO NPs Treatment

**Figure 4:**
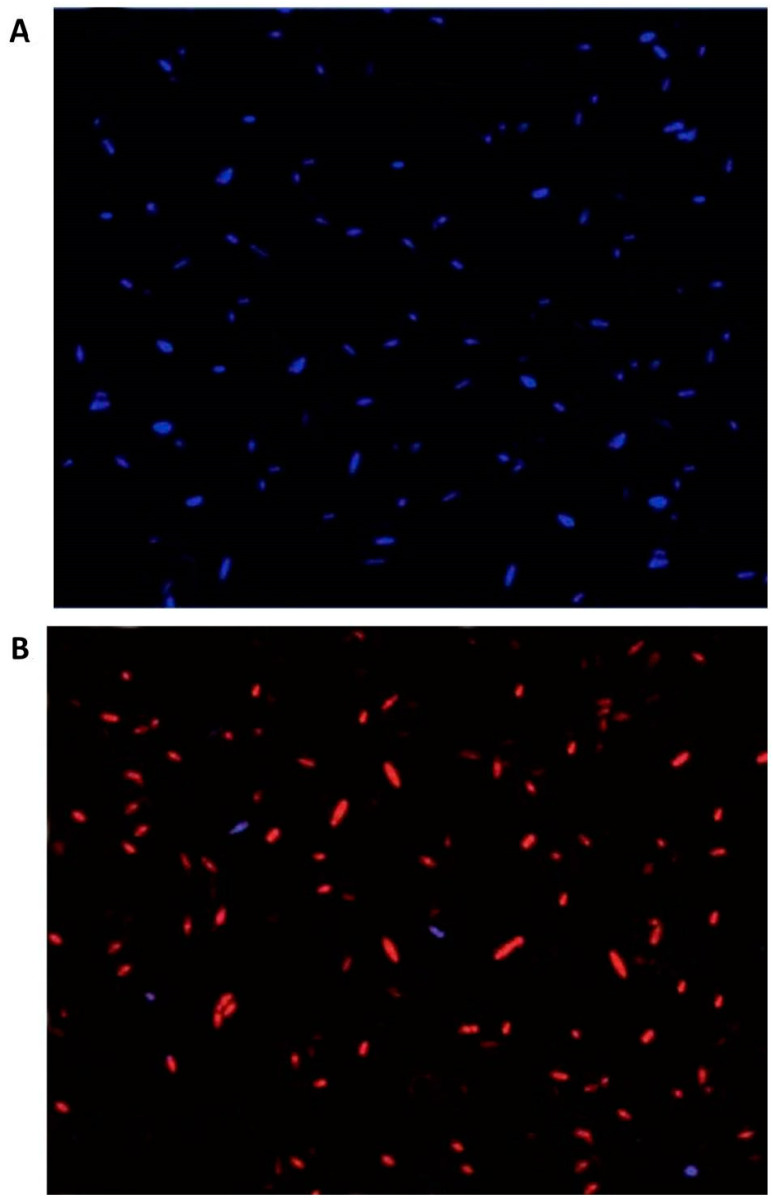
Analysis of fungal cell viability by fluorescence microscopy. *F. oxysporum* spores (**A**) non-treated or (**B**) treated with rGO-CuO NPs were stained with DAPI (stains live spores in blue) and PI (stains dead spores in red) and visualized.

The antifungal activity of rGO-CuO NPs against the FORL isolate was also analyzed under the fluorescence microscope by using PI and DAPI as fluorescent dyes. While DAPI penetrates the living cells with intact membrane and strongly binds to nucleic acids emitting blue fluorescence, PI dye is membrane-impermeable and exclusively labels broken cells with red fluorescence emission [40,41]. A culture of *F. oxysporum* FORL isolate was treated with 1 mg/L rGO-CuO NPs (5-nm size). Spores from the treated fungal culture were stained with DAPI and PI, and observed under a fluorescence microscope. While spores from the untreated control culture exclusively showed blue fluorescence (Figure 4A), spores from the rGO-CuO NP-treated culture mainly exhibited the PI red fluorescence, indicating cell death (Figure 4B).

### 3.6. Microscopic Analysis of the Interaction between rGO-CuO NPs and Fungal Cells

**Figure 5:**
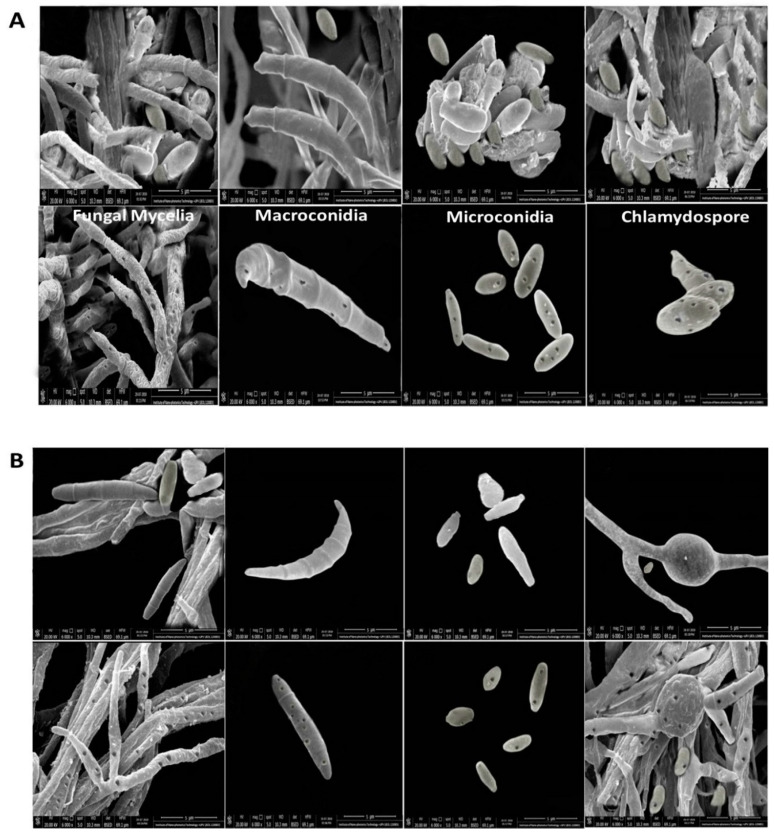

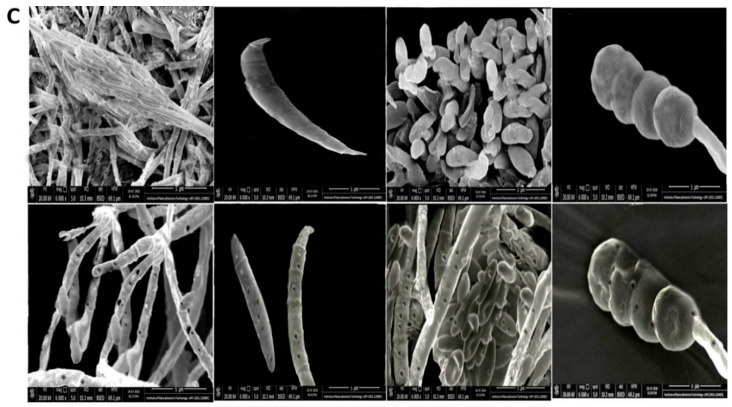
Effect of rGO-CuO NPs at 1 mg/L concentration on (**A**) FORL, (**B**) FOC1 and (**C**) FOC2. SEM images of mock-treated (upper rows) and NP-treated (lower row) fungi. Pictures in the different columns correspond to fungal mycelia, macroconidia, microconidia, and chlamydospores, as indicated.

To further explore the interaction among rGO-CuO NPs and *F. oxysporum*, hyphae, macroconidia, microconidia, and chlamydospores from the three fungal isolates treated with 1 mg/mL of 5-nm rGO-CuO NPs were imaged by HR-SEM. In contrast to the untreated controls (Figure 5A–C, upper rows), SEM images showed dense rGO-CuO NPs aggregates on the outer layer of the fungal cells in all treated isolates (Figure 5A–C, lower rows). A number of irregular pits and pores could be observed on the cell wall surface of the hyphae, macroconidia, microconidia and chlamydospores of all three treated fungal isolates (Figure 5). The presence of pores and irregular cavities on the cell wall surface must cause significant damage. As illustrated in other studies, GO nanosheets tend to non-specifically wrap the microbial cells [41]. It was also suggested that CuO NPs might be interacting with molecules containing P and S either outside or inside the fungal cells [42]. Positively charged rGO-CuO NPs may bind to negatively charged fungal membranes, and this electrostatic attraction may cause disruption in the fungal cell wall functions with damage in the protein synthesis profile and the intracellular ion efflux, and finally resulting in fungal cell death [40]. Spores have been also shown to become deformed as a result of their intertwining by the thin GO sheets [43,44,45].

**Figure 6:**
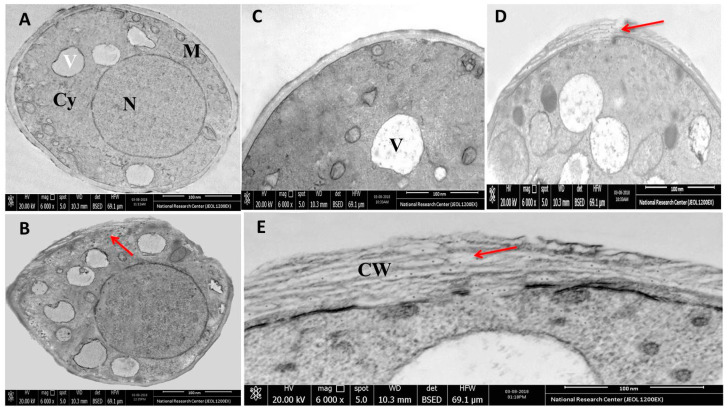
Ultra structural images obtained by TEM of (**A**,**C**) untreated and (**B**,**D**,**E**) rGO CuO NP-treated FORL cells. (**E**) Magnified view for treated fungal cell wall layers showing the internalization of NPs and their distribution. Red arrows point successful penetration of the rGO-CuO NPs in the fungal cells.

Next, we further investigated the antifungal activity of rGO-CuO NPs by TEM analysis. While spores treated with H_2_O, as a control, exhibited regular subcellular organelles and structures, including a smooth cell wall surface and an undamaged cyto-architecture (Figure 6A,C), in spores incubated for 1 h in 1 mg/L of rGO-CuO NPs, the nanocomposite was adsorbed onto the fungal cell wall surface (Figure 6B), and passed through the fungal cell wall layers, being distributed in the cell cytoplasm and also interacting with all cytoplasmic components (Figure 6D,E), which may be related to a change of cell permeability [43]. 

These results are in agreement with our previous findings showing the action of Ag NPs on fungal cell organelles and DNA in *Alternaria solani* [46]. Moreover, since rGO and CuO themselves have a large affinity to molecules rich in S and P ions, different reports suggested that either S containing proteins in the fungal cell membrane, or P containing biomolecules such as nucleic acids might be the target sites of NPs inside the cell [28,44,47,48,49,50].

### 3.7. rGO-CuO NPs Effectiveness against Fusarium Diseases

**Figure 7:**
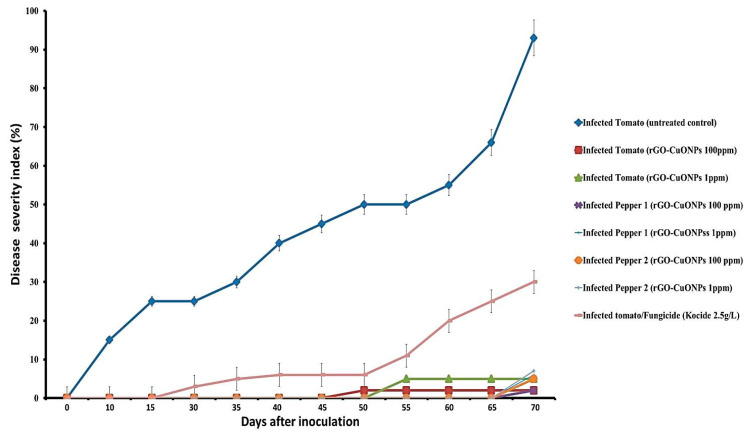
Disease severity index in % of tomato and pepper plants non-treated, treated with rGO-CuO NPs or the conventional fungicide Kocide and, then, challenged with *F. oxysporum*. Average and standard error of nine biological replicates are shown.

**Figure 8:**
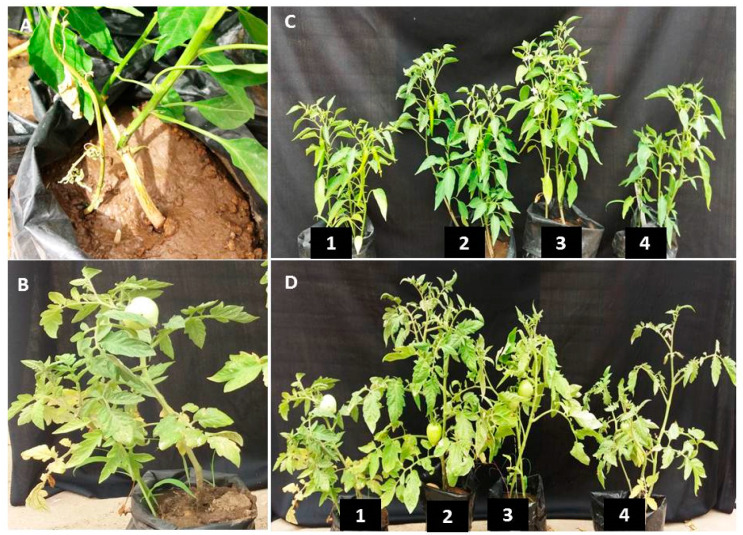
In vivo treatments of tomato and pepper plants with rGO-CuO NPs. (**A**) Pepper plant showing Fusarium wilt symptoms. (**B**) Infected control of tomato plant. (**C**,**D**) Pepper (**C**) and tomato (**D**) plants non-treated (**1**) or treated with 1 mg/L (**2**) or 100 mg/L rGO-CuO NPs (**3**), or treated with Kocide (**4**). Pictures were taken at 60 dpi.

The antifungal activity of rGO-CuO NPs was further investigated in experiments in vivo aimed to control Fusarium wilt and root rot diseases affecting tomato and pepper plants. Disease severity was monitored for 70 dpi in tomato and pepper plants treated with 1 or 100 mg/L of rGO-CuO NPs. The experiment also included plants treated with a conventional fungicide. In mock-treated tomato plants, disease symptoms were clearly observed as soon as 10 dpi, whereas in tomato treated plants, disease symptoms were slight, and largely delayed (Figure 7). Disease symptoms in pepper plants were even more delayed (Figure 7). Control treatment of tomato plants with a conventional fungicide (Kocide) also delayed and reduced disease symptoms, but at a lesser extent than rGO-CuNPs (Figure 7). No substantial differences were observed in plants subjected to 1 or 100 mg/L of rGO-CuNPs (Figure 7). Figure 8 shows pictures of representative tomato and pepper plants, mock-treated, treated with 1 or 100 mg/L of rGO-CuO NPs, or treated with Kocide, and then transplanted to infectious soil. Tomato and pepper plants treated with 1 or 100 mg/L of rGO-CuO NPs showed a healthier aspect than mock-treated or even plants treated with Kocide at 60 dpi (Figure 8C,D).

Importantly, a really high reduction in disease severity of both wilt and root rot was observed in tomato and pepper plants that were immersed for 1 h in rGO-CuO NP solution before transplanting to infectious soil. Disease severity was reduced 98 and 95% in tomato plants treated with 1 and 100 mg/L rGO-CuO NPs, respectively, and 96.5 and 93.5% (as an average of two independent experiments), respectively, in pepper plants also treated with the same amounts of NPs. Importantly, rGO-CuO NPs at either 1 or 100 mg/L did not produce any apparent phytotoxicity. These results indicated that rGO-CuO NPs, at the very low concentration of 1 mg/L, exhibit better antifungal activity than the chemical fungicide Kocide. This finding is in agreement with previous reports, in which CuO NPs performed superiorly to other six metallic oxide nanoparticles (ZnO, TiO, AlO, FeO, NiO, or MnO) in their efficiency to improve growth of tomato and eggplants grown in soil infected with Verticillium dahliae and *F. oxysporum* f.sp. *lycopersici*, respectively [48,51].

In this regard, some reports have even indicated a wide range of antimicrobial effects on pathogenic bacteria and fungi in plants for GO [23]. Furthermore, Ocsoy et al. prepared DNA-directed Ag NPs grown on GO surface, which productively controlled bacterial spots in tomatoes [15]. In addition, they detailed that GO had a positive effect on seed germination and seedling development in tomatoes. However, our work importantly shows that a single application of CuO NPs delivered on rGO nanosheets at very low concentration (1 mg/L) was only required to secure season-long pathogen suppression and yield enhancement. Beside to the unique hydrophilic reactive oxygen functional groups on the surface, GO easily disperses in water [39]. One possible explanation for the long lasting benefits of rGO-CuO NPs could be that Fusarium root infection occurs on young seedling plants early in the season [51,52], highlighting the importance of a disease/treatment window. If plant root cells have sufficient rGO-CuO NP availability, host defense may inhibit or reduce the Fusarium infection significantly and also delay the onset of Fusarium wilt disease symptoms to the extent that disease does not significantly take hold. Another possible mechanism is that rGO-CuO NPs might up-regulate host defense related genes. This would help tomato and pepper plants to cope with Fusarium infection. Although our observations are promising, they do not definitely decipher the exact mechanism by which rGO-CuO NPs control the fungal disease. However, it was proposed previously that rGO-CuO NPs are able to pass through root cells and translocate into other parts of the treated plants [51], thus inducing systemic resistance.

### 3.8. Effect of rGO-CuO on Flower Induction and Photosynthetic Pigments

**Figure 9:**
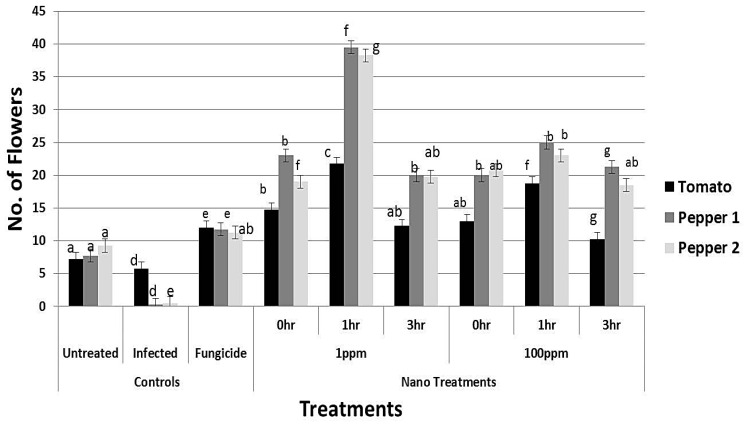
Effect of rGO-CuO NP treatments, as indicated, on flower increment in tomato and pepper plants (nine biological replicates). Two independent experiments with pepper plants are plotted. Letters on bars mean statistically significant differences between the mean of the respective control and the nanocomposite at the same concentration (*p* ˂ 0.05).

**Figure 10:**
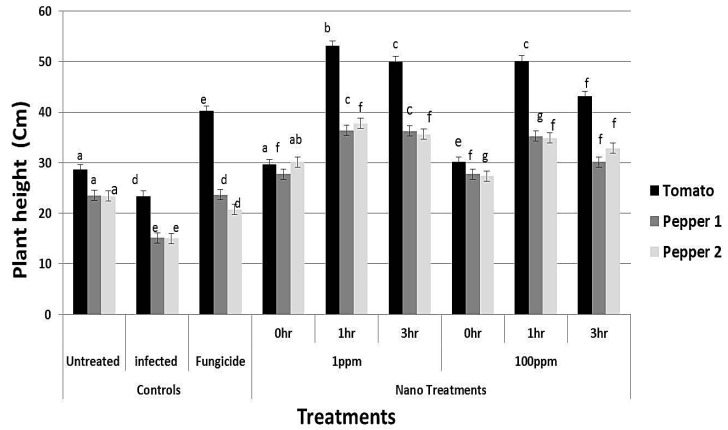
Effect of rGO-CuO NP treatments, as indicated, on plant height of tomato and pepper plants (nine biological replicates). Two independent experiments with pepper plants are plotted. Letters on bars mean statistically significant differences between the mean of the respective control and the nanocomposite at the same concentration (*p* ˂ 0.05).

**Figure 11:**
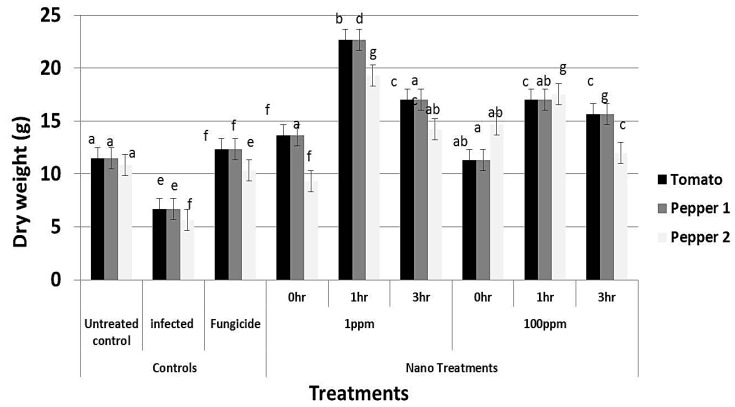
Effect of rGO-CuO NP treatments, as indicated, on dry weight of tomato and pepper plants (nine biological replicates). Two independent experiments with pepper plants are plotted. Letters on bars mean statistically significant differences between the mean of the respective control and the nanocomposite at the same concentration (*p* ˂ 0.05).

**Figure 12:**
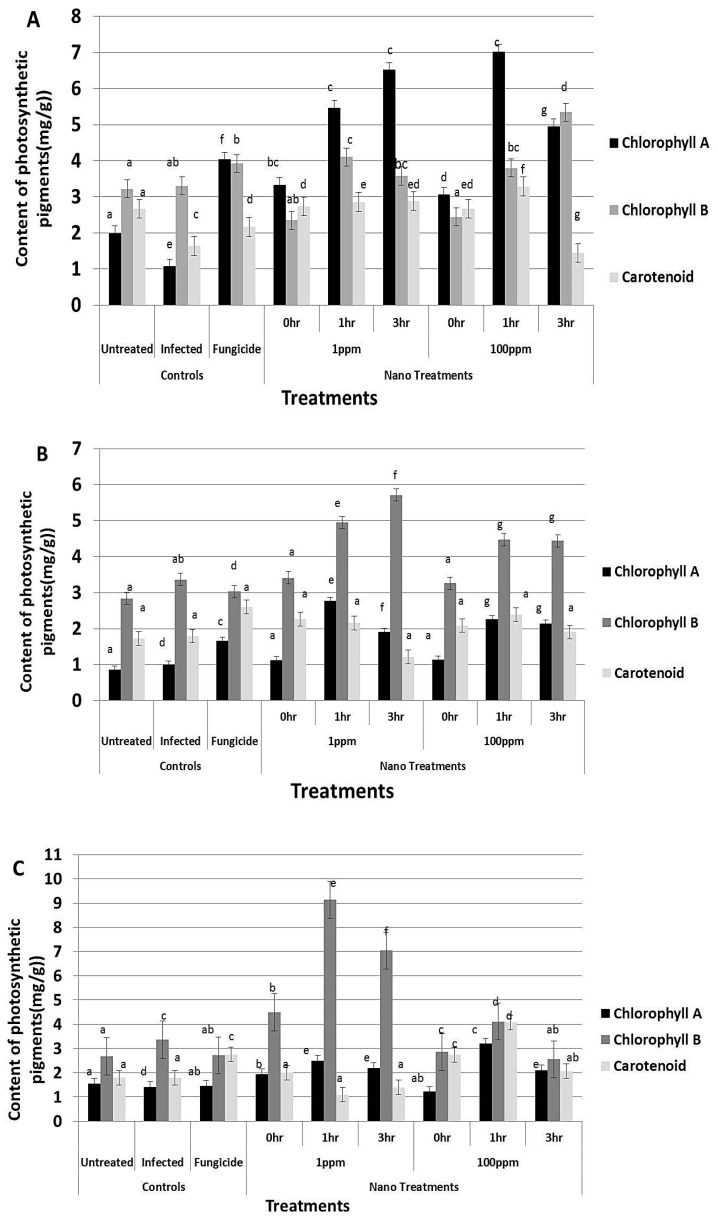
Effect of rGO-CuO NP treatments on photosynthetic pigments of treated (**A**) tomato and (**B**,**C**) pepper plants (nine biological replicates). Two independent experiments with pepper plants are plotted. Letters on bars mean statistically significant differences between the mean of the respective control and the nanocomposite at the same concentration (*p* ˂ 0.05).

Application of rGO-CuO NPs to tomato and pepper plants also improved or induced early flowering, which positively reflected on increment in the crop yield. A significant increment in the number of flowers was observed in all treatments (Figure 9). Application of NPs at both concentrations (1 and 100 mg/L) significantly induced early flowering set in tomato and pepper plants when compared to the fungicide treatment (Figure 9). Similarly, a noticeable increase in both plant height and dry weight of all NP-treated plants was observed when compared to conventional fungicide-treated plants (Figure 10 and Figure 11). In sum, a significant positive behavior was observed for plants whose roots where immersed in the NP solution. Optimal immersion time was 1 h (at both rGO-CuO NP concentrations). These results are in agreement with data published by [52]. Interestingly, it was also observed that photosynthetic pigments were also significantly increased in all NP treatments in comparison with treatment with the conventional chemical fungicide (Figure 12). These results indicate that the 5-nm rGO-CuO NPs significantly improve the levels of photosynthetic pigments in treated plants, which is in contrast to previous results, in which a dose dependent reduction in fresh weight, root length, and total chlorophyll was reported [53]. However, in that study, authors used free CuO NPs of a larger size (30 nm). Taken together, all these results indicate, that CuO NPs loaded on rGO nanosheets play a dual functional role in inducing defense response to protect the treated plants from wilt and root rot diseases, as well as promoting the plant growth. These findings may be related to their unique physicochemical properties including their stability, small size and water solubility, which endow these NPs with the potential to enter into the plant system easily in comparison to other essential nutrients.

### 3.9. Translocation of CuO NPs in Plant Roots and Potential Phytotoxicity

**Figure 13:**
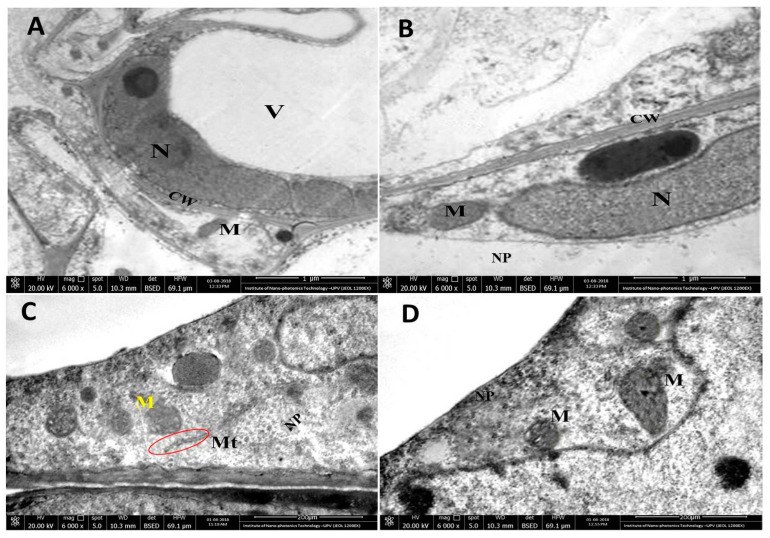
TEM micrographs showing internalization and translocation of rGO-CuO NPs (NP) in tomato root cells upon exposure to 100 mg/L of NP aqueous suspensions for 1 h. (**A**) Control (untreated) tomato root cells showing normal architecture of cell wall (Cw), nucleus (N), integrated mitochondria (M), and vacuoles (V). (**B**) Tomato treated root cells showing normal cell organelles after translocation of NPs inside cellular tissue. (**C**) Magnified view of mitochondrion (M), microtubules (Mt), and cellular matrix of tomato treated cells. (**D**) Magnified view of mitochondrion (M) and cellular matrix of pepper treated. B-D panels show internalization of NPs at various sites of root cells.

Priester et al. showed that, after uptake, CuO NPs move into different plant tissues such as stem, leaves, and fruits in tomato plants. NPs were distributed in different cellular compartments such as plant cell wall, cytoplasm, stellar system, lipid envelope and vacuoles [48]. To further analyze the possible phytotoxicity of rGO-CuO NPs, we performed TEM analysis of treated tissues. The ultrastructure analyses clearly indicated a successful penetration and crossing of the cell membrane by those nanoparticles and also demonstrated their translocation in the root cells of the treated plants. The obtained results also indicated that CuO NPs not only were found in the periplasmic spaces of confluent parenchymal cells but also had the potential to translocate into the plant cells without causing any deformation in the cell organelles (Figure 13). Interestingly, the TEM images showed clear healthy plant cellular organelles, and also the structures of cell nucleus in root system were noticed healthy clear. Furthermore, the chromatin structure was homogeneously distributed in the nucleus. Moreover, the nucleolus was complete and the boundary to nucleoplasm was distinct without any impurities; mitochondria were spherical or oval shaped and the cell double membrane was impassive, with apparent cristaes; the vacuole was well shaped. Also, the morphological structure of the plant cell wall was noticed complete and the surface was not harmed being smooth in nature, without any aggregation of NPs. These results agree with different reports showing that different edible plants uptake CuO NPs without causing any harmful effect [15,54].

## 4. Conclusions

The present research aimed to investigate the antifungal activity of rGO loaded with CuO NPs against three pathogenic strains of *F. oxysporum* that infect tomato and pepper plants. First, rGO-CuO NPs were synthesized and characterized by means of spectroscopic and microscopic analyses. Then, the antifungal properties of the nanocomposite were confirmed by in vitro growth assays with the three fungal isolates. The lethal effect of rGO-CuO NPs on fungal spores was confirmed by fluorescence analysis. Ultra structural analysis of fungal tissue suggested possible mechanisms of action. Importantly, in vivo assays with tomato and pepper plants affected by wilt and root rot diseases, in which a conventional chemical fungicide was used for comparative purposes, showed superior antifungal activity of rGO-CuO NPs and a long lasting effect at the very low concentration of 1 mg/mL. Interestingly, as CuO is a plant nutrient, analysis of treated plants indicated a beneficial effect on flowering, plant height and dry weight, as well as accumulation of photosynthetic pigments. Finally, no signs of phytotoxicity were observed by ultrastructural analysis of tissues from treated plants. In conclusion, rGO-CuO NPs is a promising nanocomposite for crop protection that shows a potent antifungal effect against *Fusarium oxysporum* and that, remarkably, has beneficial side-effects on plant growth.

## Figures and Tables

**Table 1:** Mean of inhibitory growth rate (%) of the pathogenic three *F. oxysporum* isolates with the smaller size (5 nm) of the rGO-CuO NPs.

Pathogen	Treatment		Concentration	Inhibition Rate (%)		
				4 Days	6 Days	8 Days
*F. oxysporum* (FORL)	rGO-CuO nanocomposite		1 mg/L	92.35 ± 2.047	93.15 ± 1.09	92.37 ± 1.35
			10 mg/L	92.90 ± 1.1	93.153 ± 1.09	93.7 ± 1.28
			100 mg/L	92.89 ± 1.13	93.17 ± 1.08	94.44 ± 0.0
	Controls	rGO	1 g/L	5.55 ± 0.0	8.33 ± 1.1	11.11 ± 0.0
		CuO NPs	1 g/L	19.22 ± 0.0	22.22 ± 0.0	33.33 ± 1.1
		Kocide 2000	2.5 g/L	65.3 ± 0.2	67.2 ± 0.4	68.3 ± 1.1
*F. oxysporum* (FOC1)	rGO-CuO nanocomposite		1 mg/L	89.06 ± 0.31	92.35 ± 0.30	92.80 ± 0.3
			10 mg/L	89.06 ± 0.31	92.36 ± 0.35	92.80 ± 0.33
			100 mg/L	89.08 ± 0.27	92.35 ± 0.35	92.80 ± 0.33
	Controls	rGO	1 g/L	2.27 ± 0.0	5.55 ± 1.1	8.33 ± 0.20
		CuO NPs	1 g/L	25.00 ± 1.0	33.33 ± 1.1	38.88 ± 1.1
		Kocide 2000	2.5 g/L	64.5 ± 0.5	69.2 ± 0.2	68.9 ± 0.4
*F. oxysporum* (FOC2)	rGO-CuO nanocomposite		1 mg/L	85.95 ± 6.30	81.24 ± 7.1	83.34 ± 3.4
			10 mg/L	87.13 ± 2.49	82.07 ± 1.8	82.13 ± 4.5
			100 mg/L	87.72 ± 2.8	88.77 ± 2.8	87.8 ± 2.5
	Controls	rGO	1 g/L	5.55 ± 0.0	8.33 ± 1.1	11.11 ± 0.0
		CuO NPs	1 g/L	19.22 ± 0.0	22.22 ± 0.0	30.55 ± 0.0
		Kocide 2000	2.5 g/L	63.3 ± 0.5	65.2 ± 0.4	70.5 ± 1.0

## Supplementary Materials

The following are available online at https://www.mdpi.com/2079-4991/10/5/1001/s1. Figure S1: The three *Fusarium oxysporum* isolates (A) FORL, (B) FOC1 and (C) FOC2. Mycelial fungal growth on PDA plates (upper row) and SEM images of macroconidia (lower row). Figure S2: Partial sequences of 5.8S ribosomal RNA, complete sequences of internal transcribed spacer 2, and partial sequences of large subunit ribosomal RNA gene for the three FORL, FOC1 and FOC2 *Fusarium oxysporum* isolates. Figure S3: Dynmic light scattering (DLS) analysis of the CuO NPs, (A) 5 nm, (B) 20 nm, and (C) 50 nm.
