# Supplementary material for: Reduced Graphene Oxide Nanosheet-Decorated Copper Oxide Nanoparticles: A Potent Antifungal Nanocomposite against Fusarium Root Rot and Wilt Diseases of Tomato and Pepper Plants
Source: Nanomaterials (Basel). 2020 May 24;10(5):1001. doi: 10.3390/nano10051001 (PMC7281300; doi:10.3390/nano10051001)
Supplement: Supplementary file 1 [file nanomaterials-10-01001-s001.pdf]

<u>**Supplementary Materials**</u>

Reduced Graphene Oxide Nanosheet-Decorated Copper Oxide Nanoparticles: A Potent Antifungal Nanocomposite against Fusarium Root Rot and Wilt Diseases of Tomato and Pepper Plants

Sozan E. El-Abeid [1], Yosra Ahmed [1], José-Antonio Daròs [2] and Mohamed A. Mohamed [1,2,3,]*

[1] Mycology and Disease Survey Research Department, Plant Pathology Research Institute, Agricultural Research Center, Giza 12619, Egypt; sozanelabeid@yahoo.com (S.E.E.-A.); yosra242@yahoo.com (Y.A.)

[2] Instituto de Biología Molecular y Celular de Plantas (Consejo Superior de Investigaciones Científicas—Universitat Politècnica de València), Avenida de los Naranjos, 46022 Valencia, Spain; jadaros@ibmcp.upv.es

[3] Nanotechnology & Advanced Nano-Materials Laboratory (NANML), Mycology and Disease Survey Research Department, Plant Pathology Research Institute, Agricultural Research Center, Giza 12619, Egypt

* Correspondence: mohammed_sharouny@yahoo.com; Tel.: +2-01018274608

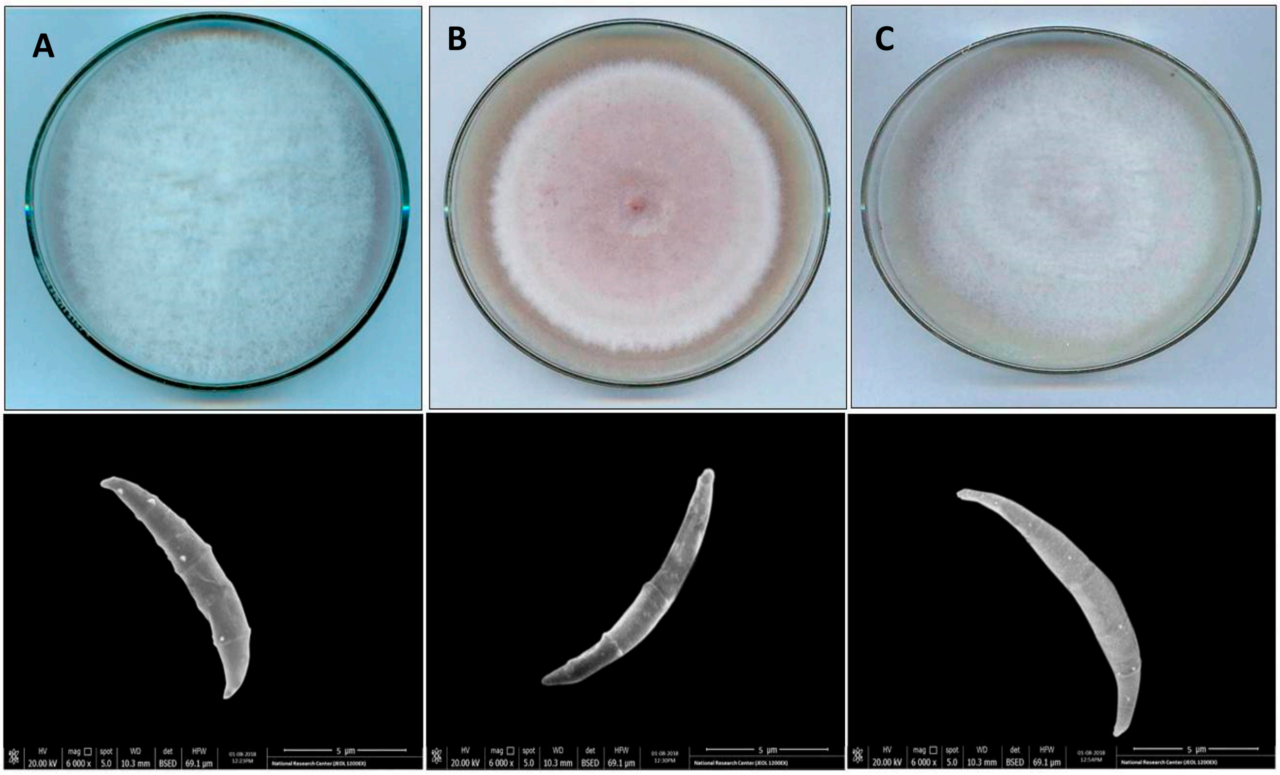

Figure S1. The three *Fusarium oxysporum* isolates (A) FORL, (B) FOC1 and (C) FOC2. Mycelial fungal growth on PDA plates (upper row) and SEM images of macroconidia (lower row).

Figure S2. Partial sequences of 5.8S ribosomal RNA, complete sequences of internal transcribed spacer 2, and partial sequences of large subunit ribosomal RNA gene for the three FORL, FOC1 and FOC2 *Fusarium oxysporum* isolates.

FORL

>MK937617.1 Fusarium oxysporum f. sp. radicis-lycopersici voucher F1 internal transcribed spacer 1, partial sequence; 5.8S ribosomal RNA gene and internal transcribed spacer 2, complete sequence; and large subunit ribosomal RNA gene, partial sequence

TGCGGAGGGATCATTACCGAGTTTACAACTCCCAAACCCCTGTGAACATACCACTTGTTGCC
TCGGCGGATCAGCCCGCTCCCGGTAAAACGGGACGGCCCGCCAGAGGACCCCTAAACTCTGT
TTCTATATGTAACTTCTGAGTAAAACCATAAATAAATCAAAACTTTCAACAACGGATCTCTT
GGTTCTGGCATCGATGAAGAACGCAGCAAAATGCGATAAGTAATGTGAATTGCAGAATTCAG
TGAATCATCGAATCTTTGAACGCACATTGCGCCCGCCAGTATTCTGGCGGGCATGCCTGTTC
GAGCGTCATTTCAACCCTCAAGCACAGCTTGGTGTTGGGACTCGCGTTAATTCGCGTTCCCC
AAATTGATTGGCGGTCACGTCGAGCTTCCATAGCGTAGTAGTAAAACCCTCGTTACTGGTAA
TCGTCGCGGCCACGCCGTTAAACCCCAACTTCTGAATGTTGACCTCGGATCAGGTAGGAATA
CCCGCTGAACTTAA

FOC1

>MK937618.1 Fusarium oxysporum voucher F2 internal transcribed spacer 1, partial sequence; 5.8S ribosomal RNA gene and internal transcribed spacer 2, complete sequence; and large subunit ribosomal RNA gene, partial sequence

TGCGGAGGGATCATTACCGAGTTTACAACTCCCAAACCCCTGTGAACATACCACTTGTTGCC
TCGGCGGATCAGCCCGCTCCCGGTAAAACGGGACGGCCCGCCAGAGGACCCCTAAACTCTGT
TTCTATATGTAACTTCTGAGTAAAACCATAAATAAATCAAAACTTTCAACAACGGATCTCTT
GGTTCTGGCATCGATGAAGAACGCAGCAAAATGCGATAAGTAATGTGAATTGCAGAATTCAG
TGAATCATCGAATCTTTGAACGCACATTGCGCCCGCCAGTATTCTGGCGGGCATGCCTGTTC
GAGCGTCATTTCAACCCTCAAGCACAGCTTGGTGTTGGGACTCGCGTTAATTCGCGTTCCTC
AAATTGATTGGCGGTCACGTCGAGCTTCCATAGCGTAGTAGTAAAACCCTCGTTACTGGTAA
TCGTCGCGGCCACGCCGTTAAACCCCAACTTCTGAATGTTGACCTCGGATCAGGTAGGAATA
CCCGCTGAACTTAA

FOC2

>MK937619.1 Fusarium oxysporum voucher F3 internal transcribed spacer 1, partial sequence; 5.8S ribosomal RNA gene and internal transcribed spacer 2, complete sequence; and large subunit ribosomal RNA gene, partial sequence

TGCGGAGGGATCATTACCGAGTTTACAACTCCCAAACCCCTGTGAACATACCACTTGTTGCC
TCGGCGGATCAGCCCGCTCCCGGTAAAACGGGACGGCCCGCCAGAGGACCCCTAAACTCTGT
TTCTATATGTAACTTCTGAGTAAAACCATAAATAAATCAAAACTTTCAACAACGGATCTCTT
GGTTCTGGCATCGATGAAGAACGCAGCAAAATGCGATAAGTAATGTGAATTGCAGAATTCAG
TGAATCATCGAATCTTTGAACGCACATTGCGCCCGCCAGTATTCTGGCGGGCATGCCTGTTC
GAGCGTCATTTCAACCCTCAAGCACAGCTTGGTGTTGGGACTCGCGTTAATTCGCGTTCCCC
AAATTGATTGGCGGTCACGTCGAGCTTCCATAGCGTAGTAGTAAAACCCTCGTTACTGGTAA
TCGTCGCGGCCACGCCGTTAAACCCCAACTTCTGAATGTTGACCTCGGATCAGGTAGGAATA
CCCGCTGAACTTAA

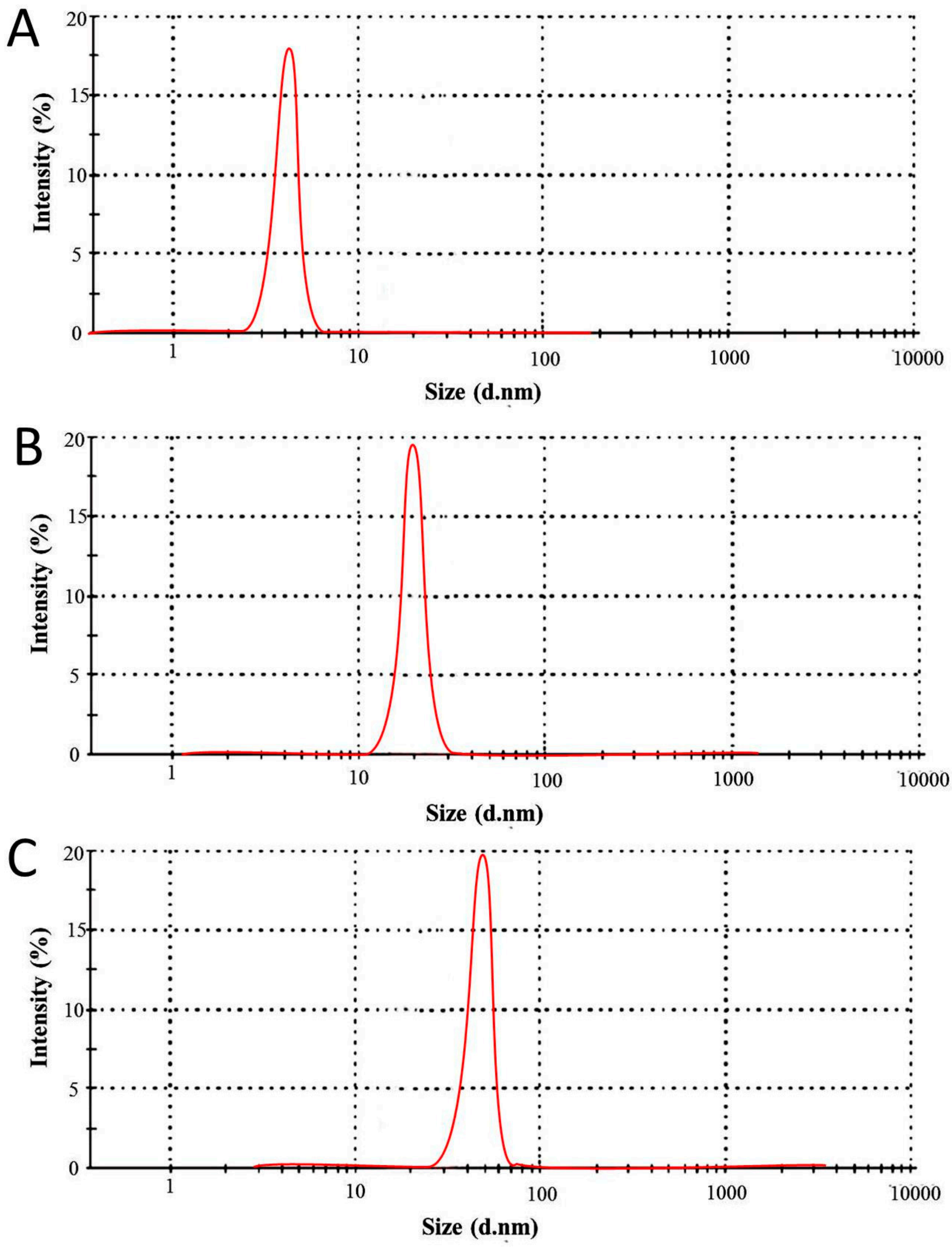

Figure S3. Dynmic light scattering (DLS) analysis of the CuO NPs, (A) 5 nm, (B) 20 nm, and (C) 50 nm.
